# Salsolinol—neurotoxic or Neuroprotective?

**DOI:** 10.1007/s12640-019-00118-7

**Published:** 2019-11-15

**Authors:** Magdalena Kurnik-Łucka, Gniewomir Latacz, Adrian Martyniak, Andrzej Bugajski, Katarzyna Kieć-Kononowicz, Krzysztof Gil

**Affiliations:** 1 Department of Pathophysiology, Faculty of Medicine, Jagiellonian University Medical College, Czysta 18, 31-121 Krakow, Poland; 2 Department of Technology and Biotechnology of Drugs, Faculty of Pharmacy, Jagiellonian University Medical College, Medyczna 9, 30-688 Krakow, Poland

**Keywords:** Salsolinol, SH-SY5Y, Wistar rats, Tetrahydroisoquinolines

## Abstract

Salsolinol (6,7-dihydroxy-1-methyl-1,2,3,4-tetrahydroisoquinoline), widely available in many edibles, is considered to alter the function of dopaminergic neurons in the central nervous system and thus, multiple hypotheses on its either physiological and/or pathophysiological role have emerged. The aim of our work was to revisit its potentially neurotoxic and/or neuroprotective role through a series of both in vitro and in vivo experiments. Salsolinol in the concentration range 10–250 μM did not show any significant release of lactate dehydrogenase from necrotic SH-SY5Y cells and was able in the concentration of 50 and 100 μM to rescue SH-SY5Y cells from death induced by H_2_O_2_. Its neuroprotective effect against neurotoxin 6-hydroxydopamine was also determined. Salsolinol was found to decrease significantly the reactive oxygen species level in SH-SY5Y cells treated by 500 μM H_2_O_2_ and the caspase activity induced by 300 μM of H_2_O_2_ or 100 μM of 6-hydroxydopamine. Serum levels of TNFα and CRP of salsolinol-treated rats were not significantly different from control animals. Both TNFα and CRP served as indirect markers of neurotoxicity and/or neuroprotection. Although the neurotoxic properties of salsolinol have numerously been emphasized, its neuroprotective properties should not be neglected and need greater consideration.

## Introduction

The tetrahydroisoquinoline skeleton is encountered in a number of bioactive compounds. Tetrahydroisoquinoline derivatives may be formed endogenously as metabolites of biogenic amines (or their precursors) as well as be delivered exogenously, directly from food. The tetrahydroisoquinoline family, widespread in nature, can be divided into compounds with catechol- and non-catechol structure. The simplest representative of non-catechol tetrahydroisoquinolines, 1,2,3,4-tetrahydroisoquinoline, occurs naturally in plants (Makino et al. 1988; Niwa et al. 1989) as well as in the brain of humans, primates, and rodents (Makino et al. 1990; Niwa et al. 1987, 1993; Yamakawa et al. 1999, Yamakawa and Ohta 1999). Catechol derivatives include, among others, salsolinol (6,7-dihydroxy-1-methyl-1,2,3,4-tetrahydroisoquinoline; molecular weight 179.219 g/mol), which is usually considered as a neurotoxin with the ability to alter the function of dopaminergic neurons and dopamine metabolism in the central nervous system. It was Maruyama et al. (1995) who first suggested that salsolinol might in fact possess both neurotoxic and neuroprotective activity. Salsolinol possesses an asymmetric center at C-1 and thus, it exists as *R* and *S* enantiomers, with the predominance of (*R*)-salsolinol in human brain tissue (Deng et al. 1997; Musshoff et al. 1999, 2000, 2003, 2005). So far, numerous hypotheses on the physiological or pathophysiological role of salsolinol have emerged, mostly with regard to salsolinol as (1) a modulator of catecholaminergic neurotransmission in the nigrostriatal pathway and possibly an etiological factor in Parkinson’s disease (PD), (2) a neuromodulator in the mesolimbic pathway related to reinforcing effects of alcohol consumption, and (3) a prolactin-releasing factor in the tuberoinfundibular pathway. Yet, its neuromodulatory role is rather poorly understood, lacks detail, and remains inconclusive.

The aim of our work was to revisit the potential neurotoxic and/or neuroprotective role of salsolinol through a series of both in vitro (SH-SY5Y and IMR-32 human neuroblastoma cell lines) and in vivo (male Wistar rats) experiments.

## Materials and Methods

### In Vitro Experiments

#### Cell Lines

SH-SY5Y (ATCC® CRL-2266™) cell line, a popular cell model for PD research (Xie et al. 2010), was purchased from ATCC (Manassas, VA, USA).

IMR-32 (ATCC® CCL-127™) cell line was provided by the Department of Oncogenomics, Academisch Medisch Centrum, Amsterdam, Holland. This cell line expresses most of the proteins of cholinergic neurons and was described as an in vitro model to study on Alzheimer’s disease (AD) (Neill et al. 1994).

The cell lines were cultured in Dulbecco’s modified Eagle’s medium (DMEM/F12) with 10% fetal bovine serum obtained both from Gibco (Carlsbad, CA, USA) at 37 °C in an atmosphere containing 5% of CO_2_.

#### Chemicals

(*RS*)-salsolinol (SAL), purity ≥ 99%, was obtained from Cayman Chemical (Ann Arbor, MI, USA). 6-Hydroxydopamine (6-OHDA), the commonly applied neurotoxin in experimental animal models of PD (Blum et al. 2001), and 2′,7′-dichlorodihydrofluorescein diacetate (DCFH-DA) were purchased from Sigma-Aldrich (St. Louis, MO, USA). Hydrogen peroxide (H_2_O_2_) was obtained from Synoptis Pharma (Warsaw, Poland).

#### Lactate Dehydrogenase Test

CytoTox-ONE™ Homogeneous Membrane Integrity Assay (LDH) was purchased from Promega (Madison, WI, USA). SH-SY5Y or IMR-32 cells were seeded in 96-well white plates with transparent bottom at a concentration of 2.5 × 10^4^ cells/well in 100 μl culture medium and cultured for 24 h to reach 70% confluence. For toxicity estimation, the 10 mM SAL stock solution in DMSO was diluted into fresh culture medium and added into the microplates at the final concentrations 10–250 μM. For neuroprotective studies, SH-SY5Y cells were preincubated first for 1 h with SAL at the final concentrations 100 and 250 μM and next 6-OHDA neurotoxin was added at final concentration of 50 μM. The IMR-32 cells were tested with SAL and 6-OHDA final concentrations 100 μM and 200 μM, respectively. After 24 h of incubation, the CytoTox-ONE™ was added to each well and cells were incubated for 10 min at room temperature. Lysis solution, provided by the manufacturer, was also added to generate a maximum lactate dehydrogenase (LDH) release control. According to the protocol provided by the manufacturer the Lysis solution was added 10 min before the measurement of the fluorescence. Next, the fluorescence was measured using a microplate reader EnSpire (PerkinElmer, Waltham, MA, USA) with an excitation wavelength of 560 nm and an emission wavelength of 590 nm. All measurements were performed in triplicate and results are shown as mean ± standard deviation (SD).

#### Caspase Activity Assay

Apo-ONE® Homogeneous Caspase-3/7 Assay was purchased from Promega (Madison, WI, USA). SH-SY5Y cells were seeded in 384-well black plates with transparent bottom at a concentration of 1 × 10^4^ cells/well in 25 μl culture medium and cultured for 24 h to reach 70% confluence. Cells were preincubated first for 1 h with SAL at the final concentration of 250 μM and next 6-OHDA or H_2_O_2_ were added at final concentrations of 100 μM and 300 μM, respectively. After 6 h of incubation, Apo-ONE® Homogeneous Caspase-3/7 Reagent was added to each well and cells were incubated for 18 h at room temperature, according to the manufacturer’s protocol. The fluorescence of the samples was measured using a microplate reader EnSpire (PerkinElmer, Waltham, MA USA) with an excitation wavelength of 500 nm and an emission wavelength of 520 nm. All measurements were performed in triplicate and results are shown as mean ± SD.

#### ROS Assay

SH-SY5Y cells were seeded in 96-well white plates with transparent bottom at a concentration of 2.5 × 10^4^ cells/well in 100 μl culture medium and cultured for 24 h to reach 70% confluence. Next, the old media were removed and cells were preincubated first with DCFH-DA 250 μM solution in HBSS buffer for 1 h. After DCFH-DA was removed, cells were washed with HBSS and incubated next for 1 h with SAL at the final concentrations of 50, 100, and 250 μM in culture media. Next, the H_2_O_2_ was added at final concentration 500 μM. After 3 h of incubation, the fluorescence was measured using a microplate reader EnSpire (PerkinElmer, Waltham, MA USA) with an excitation wavelength of 486 nm and emission wavelength of 530 nm. All measurements were performed in triplicate and results are shown as mean ± SD.

#### MTS Assay

The CellTiter 96® AQueous Non-Radioactive Cell Proliferation Assay (MTS) was purchased from Promega (Madison, WI, USA). SH-SY5Y cells were seeded in 96-well transparent plates at a concentration of 2.5 × 10^4^ cells/well in 100 μl culture medium and cultured for 24 h to reach 70% confluence. The cells were preincubated first for 1 h with SAL at the final concentrations 50 and 100 μM and next H_2_O_2_ was added at final concentration 300 μM. After 24 h of incubation, the MTS labeling mixture was added to each well and cells were incubated under the same conditions for 5 h. The absorbance was measured using a microplate reader EnSpire (PerkinElmer, Waltham, MA USA) at 490 nm. All measurements were performed in triplicate and results are shown as mean ± SD.

### In Vivo Experiments

Adult male Wistar rats (Jagiellonian University Medical College Animal Laboratory, Krakow, Poland) were housed in individual transparent cages placed adjacent to each other to provide sight, acoustic, and odor contact, with food and water available ad libitum, temperature maintained at 23 ± 2 °C and under a 12:12-h dark/light cycle. Rats were fed a standard diet (protein 25%, fat 8%, carbohydrates 67%, 2.86 kcal/g, Labofeed B, Kcynia, Poland). After acclimatization period, animals were randomly divided into the following groups (*n* = 6 rats in each group): (1) continuous dosing of SAL (purity ≥ 99%, Cayman Chemical, Ann Arbor, MI, USA)—200 mg/kg in total with ALZET osmotic mini-pumps (delivery rate 0.25 μL/h, Durect, USA) implanted intraperitoneally (i.p.) for 4 weeks (S1 group); (2) continuous dosing of SAL—300 mg/kg in total with ALZET osmotic mini-pumps implanted i.p. for 4 weeks (S2 group); (3) a control group (C group). SAL was dissolved in 0.9% NaCl. Control rats received 0.9% NaCl. Mini-pumps were implanted under general anesthesia induced with sodium pentobarbital given i.p. at a dose of 0.25 mg/kg (Vetbutal, Biowet, Poland). All animal experiments were approved by the Jagiellonian University Bioethical Committee (protocol number—67/2009) and conducted in accordance with Good Laboratory Practices.

General health status and motor function of the experimental animals were evaluated daily during handling and by observing their in-cage behavior, including food intake and body weight measurements. At the end of the experiment, following 12 h overnight fasting, animals were euthanized via decapitation and blood samples from the jugular vessels were collected in plastic tubes and incubated at least 30 min at 4 °C to induce clot formation. After centrifugation at 1500×*g* for 20 min at 4 °C (Megafuge 1.0R, Heraeus Instruments, Germany), serum samples were collected and kept frozen at − 80 °C until further analysis. Serum samples were assayed for tumor necrosis factor–alpha (TNFα) (R&D Systems Europe, Ltd. UK) and C-reactive protein (CRP, eBioscience, Affymetrix, CA, USA) by ELISA method according to manufacturer’s instructions. All measurements were performed in duplicate and results are shown as mean ± SD.

### Statistical Analysis

Statistical significances of in vitro data were evaluated by GraphPad Prism™ software (version 5.01, San Diego, CA, USA) using a one-way ANOVA, followed by Bonferroni’s comparison test and are expressed as the mean ± standard deviation (SD). In vivo results were analyzed by GraphPad Prism™ software (version 7.0a, San Diego, CA, USA) using a one-way analysis of variance (ANOVA) followed by a post hoc Tukey’s test and are expressed as the mean ± SD. Statistical significance was set at *p* < 0.05.

## Results

### In Vitro Tests—Cell Cultures (SH-SY5Y, IMR-32) Together with Biochemical Assays (LDH Test, Caspase Activity, ROS, and MTS)

**Fig. 1:**
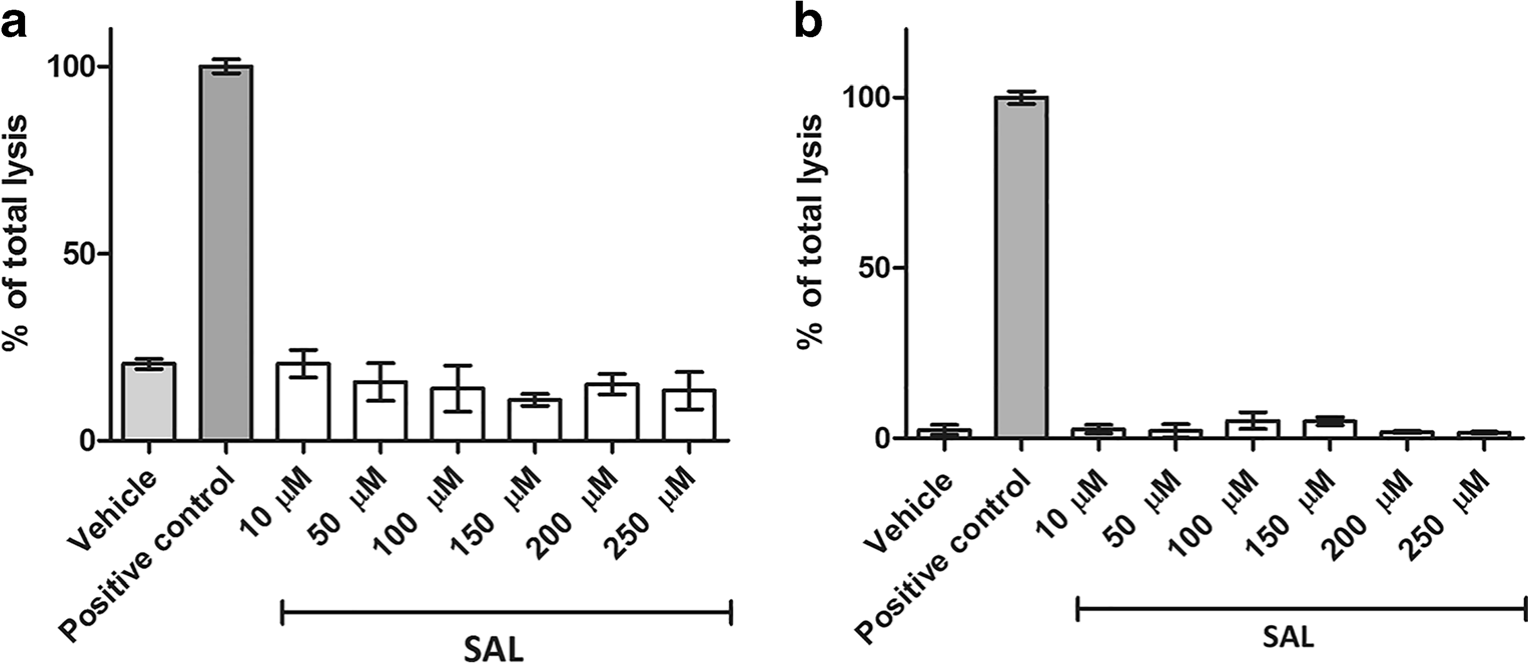
Effect of (*RS*)-salsolinol (SAL) on lactate dehydrogenase (LDH) release in SH-SY5Y neuroblastoma cells (**a**). Effect of (*RS*)-salsolinol (SAL) on lactate dehydrogenase (LDH) release in IMR-32 neuroblastoma cells (**b**)

The toxicity of SAL against human dopaminergic neuroblastoma SH-SY5Y (Xie et al. 2010) as well as cholinergic neuroblastoma IMR-32 (Neill et al. 1994) cell lines was examined first by the LDH assay. The 24 h incubation of cells with (*RS*)-salsolinol (SAL) in the concentration range 10–250 μM did not show any statistically significant increased release of LDH from necrotic cells (Fig. 1a, b) in comparison with the negative (vehicle, 1% DMSO in cell culture media) and positive (total lysis reagent) controls.

**Fig. 2:**
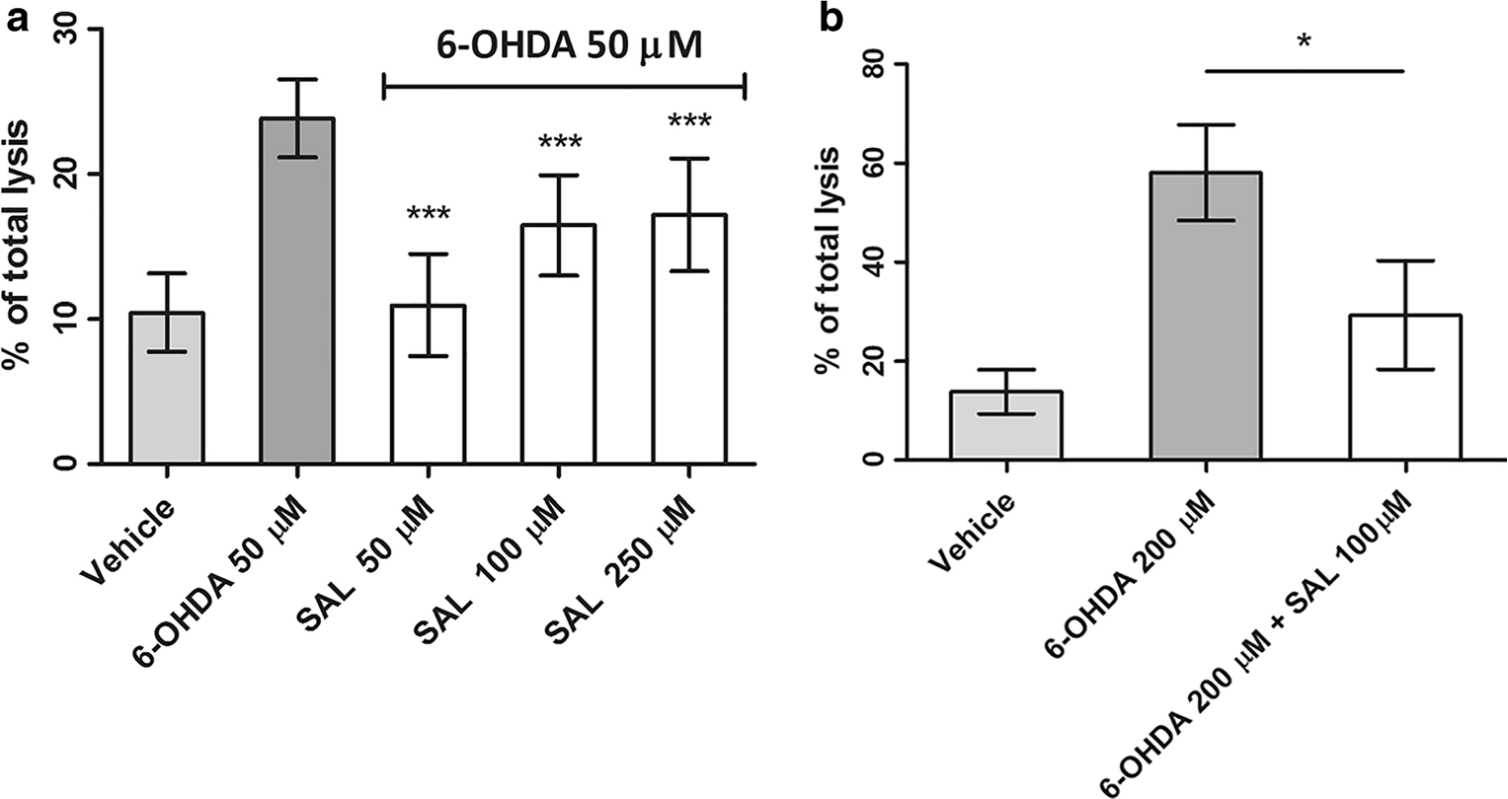
The effect of (*RS*)-salsolinol (SAL) on lactate dehydrogenase (LDH) release in SH-SY5Y neuroblastoma cells damaged by 50 μM 6-hydroxydopamine (6-OHDA) at 24 h (**a**). The effect of (*RS*)-salsolinol (SAL) on lactate dehydrogenase (LDH) release in IMR-32 neuroblastoma cells damaged by 200 μM 6-hydroxydopamine (6-OHDA) at 24 h (**b**). Statistical significance was set at ∗∗∗*p* < 0.001 and ∗*p* < 0.05 in comparison with the positive controls (6-OHDA 50 μM for SH-SY5Y cells and 6-OHDA 200 μM for IMR-32 cells)

The studies on potential neuroprotective activity of SAL were performed next with use of 6-OHDA. The SH-SY5Y cells were exposed to 6-OHDA at the concentration 50 μM for 24 h with or without the presence of SAL in various concentrations (Fig. 2a). As result, the statistically significant (*p* < 0.001) decrease in LDH release in 6-OHDA injured cells was observed in cells treated simultaneously by SAL in all tested concentrations. Similarly, SAL (100 μM) protected significantly (*p* < 0.05) IMR-32 cells exposed to 200 μM of 6-OHDA for 24 h (Fig. 2b).

**Fig. 3:**
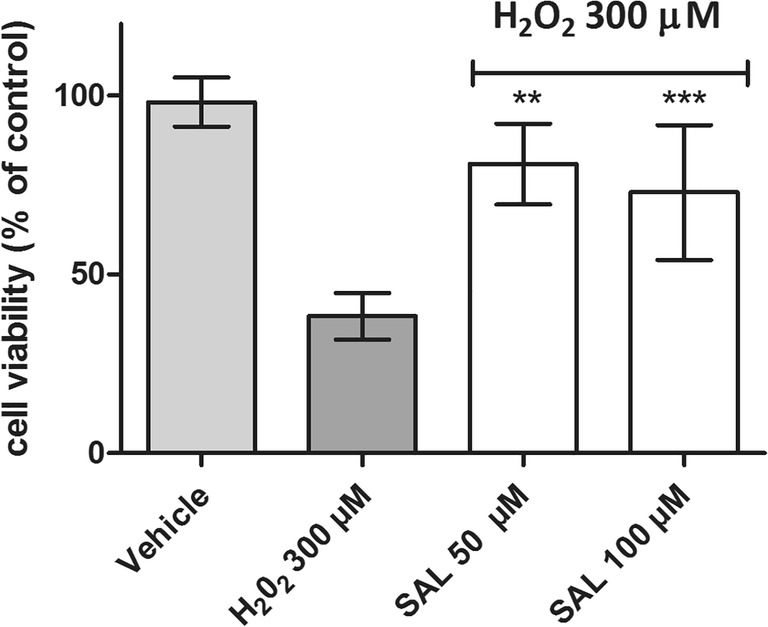
The effect of (*RS*)-salsolinol (SAL) on SH-SY5Y neuroblastoma cells viability damaged by 300 μM H_2_O_2_ at 24 h. Statistical significance was set at ∗∗∗*p* < 0.001, ∗∗*p* < 0.01, in comparison with the positive control H_2_O_2_ (300 μM)

To confirm SAL neuroprotection properties, additional studies were performed for SH-SY5Y cells with use of 300 μM of H_2_O_2_ as toxic agent. The cell viability was measured colorimetrically using standard MTS procedure. SH-SY5Y cells were incubated with H_2_O_2_ in the presence of SAL at 50 and 100 μM for 24 h. As shown in Fig. 3, MTS assay showed statistically significant (*p* < 0.01, *p* < 0.001) increase in viability of cells treated with H_2_O_2_ and SAL in comparison with the cells treated H_2_O_2_ alone (positive control).

**Fig. 4:**
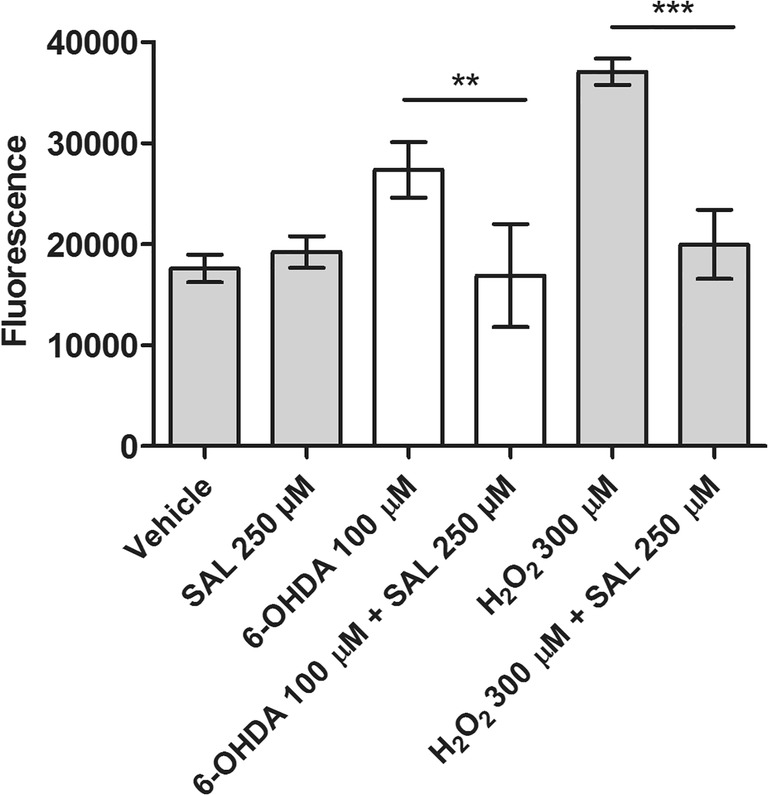
The effect of (*RS*)-salsolinol (SAL) on the caspases 3/7 activity in SH-SY5Y neuroblastoma cells damaged by 100 μM 6-hydroxydopamine (6-OHDA) at 6 h. Statistical significance was set at ∗∗*p* < 0.001 in comparison with the positive control (6-OHDA 100 μM), and ∗∗∗*p* < 0.001 in comparison with the positive control (H_2_O_2_ 300 μM)

The SAL capability to reduce the ROS level and caspase activity in cells injured by toxins was estimated next to elucidate its potential mechanism of neuroprotection. To measure the effect of SAL on caspases 3/7 expression in 6-OHDA-induced apoptosis, SH-SY5Y cells were incubated with 6-OHDA (100 μM) for 6 h. Cytoplasmic caspase-3/7 activity was then measured fluorometrically using the Apo ONE® Homogeneous Caspase-3/7 Assay. The cells treated with 6-OHDA and SAL at 250 μM showed statistically significant (*p* < 0.01) reduction in 6-OHDA-induced caspase activity (Fig. 4). The similar test was performed with use of 300 μM of H_2_O_2_ as an apoptosis inductor. The statistically significant (*p* < 0.001) reduction in H_2_O_2_-induced caspase-3/7 activity in the presence of SAL at 250 μM was also observed (Fig. 4).

**Fig. 5:**
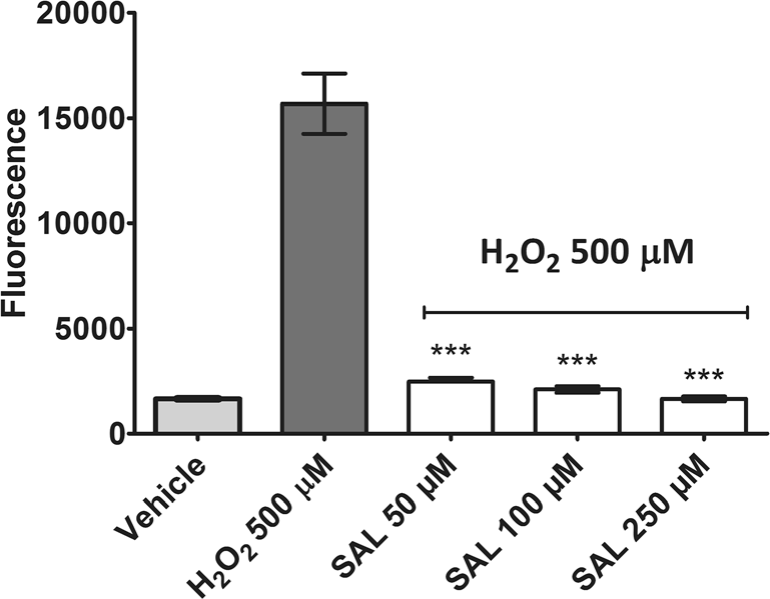
The effect of (*RS*)-salsolinol (SAL) on ROS level in SH-SY5Y neuroblastoma cells treated by 500 μM H_2_O_2_ at 3 h. Statistical significance was set at ∗∗∗*p* < 0.001, in comparison with the positive control H_2_O_2_ (500 μM)

To examine the effect of SAL on intracellular ROS reduction, the SH-SY5Y cells were incubated with H_2_O_2_ 500 μM for 3 h. The ROS level was measured using 2′,7′-dichlorodihydrofluorescein diacetate (DCFH-DA), which in the presence of ROS is oxidized to the highly fluorescent dichlorofluorescein (DCF). As shown in Fig. 5, cells treated with SAL at 50, 100, and 250 μM concentrations showed statistically significant (*p* < 0.001) reduction in ROS level (up to the negative control level) induced by 500 μM H_2_O_2_.

### In Vivo Results—Behavioral Observations (General Health Status, Motor Function, Food Intake and Body Weight Measurements) Together with Biochemical Analysis (TNFα and CRP)

None of the SAL-treated or control animals died or showed any visible disturbances or discomfort to any significant degree. No disturbance of gross motor function was observed in any of the SAL-treated rats.

The total weight gain as well as the total weight gain over the initial body weight was indistinguishable between the SAL-treated groups; however, the total food intake was slightly diminished in SAL-treated animals in comparison with the control group of rats (Table 1).

**Table 1:** Total weight gain (g), total weight gain over the initial body weight (%), and total food intake (g) in (*RS*)-salsolinol (SAL)-treated (S1, S2) and the control animals (C)

Group	Total weight gain (g)	Total weight gain over initial body weight (%)	Total food intake (g)
S1	98.34 ± 31.61	39.4 ± 15.09	644.3 ± 56.9 (*p* < 0.05)
S2	103.25 ± 8.22	42.5 ± 4.6	585.0 ± 23.5 (*p* < 0.01)
C	120.36 ± 20.21	47.7 ± 12.7	696.9 ± 40.2

Statistical significance was set at ∗*p* < 0.05

**Fig. 6:**
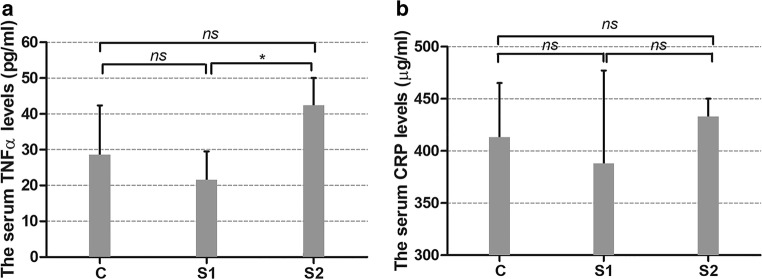
TNFα (**a**) and CRP (**b**) serum levels in (*RS*)-salsolinol (SAL)-treated (S1, S2) and control animals (C). Statistical significance was set at ∗*p* < 0.05. “*Ns*” refers to the lack of statistical significance

TNFα serum levels were only significantly different between S1 and S2 groups (21.65 pg/ml ± 7.9 vs. 42.43 pg/ml ± 7.6, *p* = 0.029; C = 25.87 pg/ml ± 11.5), but were indistinguishable in SAL-treated rats in comparison with the control group of rats. Changes in serum CRP levels were indistinguishable in all SAL-treated groups in comparison with control animals and showed similar trends to TNFα serum levels (S1 = 366 μg/ml, S2 = 555 μg/ml vs. C = 470.9 μg/ml) (Fig. 6). Both TNFα and CRP served as indirect markers of neurotoxicity and/or neuroprotection and their use is justified by several publications (Barcia et al. 2005; Tarrant 2010; Sawada et al. 2015; Umemura et al. 2015; Qiu et al. 2019).

## Discussion

Tetrahydroisoquinolines, including (*RS*)-salsolinol, are widely distributed in the environment and can also be easily formed endogenously from biogenic amines or amino acids. (*RS*)-salsolinol is available in nano- to micromolar amounts per gram in many edibles, such as fungi, vegetables, fruits, eggs, and dairy or alcohol beverages (Collins et al. 1990; DeCuypere 2010; Deng et al. 1997; Duncan et al. 1984; Duncan and Smythe 1982; Melzig et al. 2000; Riggin et al. 1976; Riggin and Kissinger 1976; Smythe and Duncan 1985; Strolin Benedetti et al. 1989a, b), see Table 2 for details.

**Table 2:** Some examples of (*RS*)-salsolinol levels in different food products

Source	Concentration range of (*RS*)-salsolinol	Analytical method	Reference
Banana	Up to 40 ± 1,5 μg/g	Liquid chromatography and thin-layer electrochemistry	(Riggin et al. 1976)
	Up to 537 μg/g	Gas chromatography with mass spectrometry	(Smythe and Duncan 1985)
	Up to 50 μg/g	High-performance liquid chromatography with electrochemical detection	(Strolin Benedetti et al. 1989a)
	709 nmol/g	Ion-pair high-performance liquid chromatography with beta-cyclodextrin	(Deng et al. 1997)
	11.22 ± 0.049 μg/g	Liquid chromatography–mass with tandem mass spectrometry	(DeCuypere 2010)
Mushroom	21.92 ± 0.136 μg/g		
Peach	14.29 ± 0.055 μg/g		
Potato	0.52 ± 0.025 μg/g		
Tomato	0.83 ± 0.049 μg/g		
Bitter chocolate	1 μg/g	High-performance liquid chromatography with electrochemical detection	(Strolin Benedetti et al. 1989b)
Cacao	Up to 25 μg/g	Gas chromatography with mass spectrometry	(Melzig et al. 2000)
Port wine	Up to 488 nmol/l		(Duncan and Smythe 1982)
	4–85 ng/ml		(Smythe and Duncan 1985)

Recently, Tuenter et al. (2018) revisited the Melzig hypothesis (Melzig et al. 2000) about the role of (*RS*)-salsolinol in the mood effects of cocoa. The D_3_-receptor is known to play a role in the reward system and (*RS*)-salsolinol (especially *S*-isomer) was found to bind to the receptor. It was hypothesized that the amount of (*RS*)-salsolinol typically ingested by consuming about 100 g of dark chocolate (with 20–25 μg/g of salsolinol racemate) was sufficient to reach a pharmacologically relevant concentration. According to Lee et al. (2010), in the long term, (*RS*)-salsolinol from different dietary sources should be the major contributor to its plasma levels, both in humans and rats. Yet, it remains unclear, whether it can cross the blood-brain barrier to exert its central action (Origitano et al. 1981; for a review, see Lee et al. 2010; Kurnik-Łucka et al. 2018). In mammalian brain, (*RS*)-salsolinol is thought to be formed via three mechanisms: (1) the nonenzymatic Pictet–Spengler condensation of dopamine and aldehydes producing salsolinol as two racemic isomers (*R* or *S*); (2) the non-enzymatic condensation of dopamine and pyruvate yielding 1-carboxyl-tetrahydroisoquinoline, followed by decarboxylation and reduction, which produces (*R*)-salsolinol; (3) selective synthesis of (*R*)-salsolinol from dopamine and acetaldehyde by (*R*)-salsolinol synthase (Naoi et al. 2002).

Numerous in vitro experiments published so far suggest that (*RS*)-salsolinol possesses mainly pro-apoptotic properties. The human dopaminergic neuroblastoma SH-SY5Y cell line model has been applied in majority of the studies with experimental doses ranging up to 2.2 mM (for a review, see Kurnik-Łucka et al. 2018). Foremostly, Morikawa et al. (1998) reported that (*RS*)-salsolinol inhibited mitochondrial complex I activity; however, its *N*-methylated derivatives or those with phenyl group at the C1 position were more toxic to NADH-linked respiration. ROS were increased and glutathione levels were decreased when SH-SY5Y cells were treated with racemic salsolinol, especially at concentrations near to 500 μM (Wanpen et al. 2004). The treatment also decreased the levels of an anti-apoptotic protein bcl-2 and increased a pro-apoptotic protein bax (Shukla et al. 2013; Wanpen et al. 2007). Storch et al. (2000) concluded that (*RS*)-salsolinol was toxic to human dopaminergic neuroblastoma SH-SY5Y cells by blocking the cellular energy supply via the inhibition of mitochondrial complex II activity but not complex I. The rapid decrease in the intracellular level of ATP and ATP/ADP ratio of intact cells incubated with (*RS*)-salsolinol was dose- and time-dependent.

But it was Maruyama et al. (1995) who suggested that (*RS*)-salsolinol and its derivatives might possess both neurotoxic and neuroprotective activity. (*R*)-salsolinol and the 1,2-dimethyl-6,7-dihydroxyisoquinolinium ion (40 and 200 μM) as well as *N*-methyl-(*R*)-salsolinol (200 μM) reduced in vivo radical formation, with reduction of dopamine catabolism. (*R*)-salsolinol and the 1,2-dimethyl-6,7-dihydroxyisoquinolinium ion also reduced in vitro hydroxyl radical production from dopamine autoxidation. On the other hand, *N*-methyl-(*R*)-salsolinol (40 μM) increased the hydroxyl radical level in the striatum, and the radical production by its autoxidation was confirmed in vitro. Only Możdżeń et al. (2015) confirmed biphasic effects of exogenous (*RS*)-salsolinol. In rat hippocampal cell cultures, the lower investigated dose of (*RS*)-salsolinol (50 and 100 μM) diminished, while its highest dose (500 μM) potentiated the glutamic acid effect on caspase-3 activity. Similar effects were observed for LDH release. In mouse striatum cultures, both the investigated doses of (*RS*)-salsolinol (50 and 500 μM) revealed its neuroprotective activity. Authors concluded that exogenous salsolinol, applied as a racemic mixture under physiological conditions, should not be neurotoxic.

Our in vitro results suggest that the possible neuroprotective properties of (*RS*)-salsolinol cannot indeed be neglected. First of all, the used in our studies highly pure (≥ 99%), (*RS*)-salsolinol in the concentration range 10–250 μM did not show any significant release of LDH from either necrotic SH-SY5Y cells (Fig. 1a) or necrotic IMR-32 cells (Fig. 1b). Moreover, a decrease in LDH release in SH-SY5Y cells damaged by 50 μM of 6-OHDA was observed when treated together with (*RS*)-salsolinol in all tested concentrations (Fig. 2a). Similarly, the presence of 100 μM of (*RS*)-salsolinol statistically significant decreased toxic activity of 200 μM of 6-OHDA against IMR-32 cells (Fig. 2b). (*RS*)-salsolinol (50 and 100 μM) was also able to rescue SH-SY5Y cells from death induced by 300 μM of H_2_O_2_ (Fig. 3). In ROS assay, SH-SY5Y cells treated with (*RS*)-salsolinol at 50, 100, and 250 μM concentrations showed a significant reduction in ROS level induced by 500 μM of H_2_O_2_ (Fig. 5). The apoptosis of SH-SY5Y cells, estimated here as the increase in caspase-3/7 activity induced by 100 μM of 6-OHDA or 300 μM of H_2_O_2_, was also reduced significantly to the control level by (*RS*)-salsolinol at 250 μM concentration. Moreover, no induction effect on caspase-3/7 activity by (*RS*)-salsolinol at 250 μM itself was found (Fig. 4).

A question arises why these results significantly differ from many others already published? The presence of two enantiomers and their origin, either endogenous or exogenous, is complex. Most of the experimental data refer to exogenous (*RS*)-salsolinol hydrochloride applied as a racemic mixture. Quintanilla et al. (2014, 2016) not only chirally separated a commercially available (*RS*)-salsolinol but also purified it from isosalsolinol. Isosalsolinol is a by-product of non-enzymatical Pictet–Spengler condensation, and at physiological pH, the reaction of acetaldehyde and dopamine generates (*RS*)-salsolinol and isosalsolinol in equal proportions, while under non-physiological in vitro conditions (low pH), the synthesis of (*RS*)-salsolinol predominates (Almodovar et al. 2017; Bates et al. 1986; King et al. 1974). Variations in methodological approaches might be a barrier to make an extensive summary. All in vitro studies should be always interpreted with caution, since extrapolation of the effects to humans is not straightforward.

**Fig. 7:**
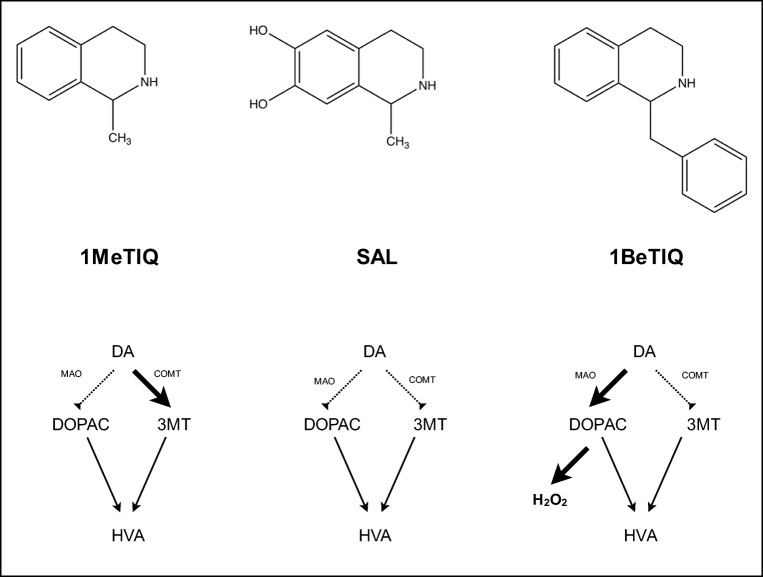
Chemical structures and the influence on the main dopamine metabolic pathways of 1MeTIQ, SAL, and 1BeTIQ. DA dopamine, DOPAC 3,4-dihydroxyphenylacetic acid, HVA homovanillic acid, SAL 6,7-dihydroxy-1-methyl-1,2,3,4-tetrahydroisoquinoline, 1BeTIQ 1-benzyl-1,2,3,4-tetrahydroisoquinoline, 1MeTIQ 1-methyl-1,2,3,4-tetrahydroisoquinoline, 3MT 3-methoxy-4-hydroxyphenethylamine

From the chemical point of view, (*RS*)-salsolinol might indeed possess neuroprotective properties due to the presence of catechol (1,2-dihydroxybenzene) moiety. The relatively high antioxidant activity of catechol can be explained by the high electron-donating effect of one hydroxyl group to the other (Fig. 7) (Heijnen et al. 2002, 2011). Indeed, the antioxidant properties of (*RS*)-salsolinol were confirmed here in vitro, as it was able to reduce the H_2_O_2_-induced increase in intracellular ROS level in SH-SY5Y cells (Fig. 5). Moreover, taking into account the possible mechanisms of 6-OHDA toxicity, the antioxidant properties of (*RS*)-salsolinol may be also responsible for determining here decrease of 6-OHDA activity (Figs. 2 and 4). The mechanism of 6-OHDA neurotoxicity was proposed to be the effect of increased level of ROS derived from autoxidation and/or obtained by the 6-OHDA biotransformation catalyzed by MAO-A (monoamine oxidase A) (Rodriguez-Pallares et al. 2007; Simola et al. 2007). Thus, also, the inhibition of MAO activity by (*RS*)-salsolinol (Fig. 7), which was first reported by Yamanaka (1971), could also contribute to its neuroprotective properties. The inhibition of 6-OHDA biotransformation catalyzed by MAO-A might decrease indirectly the intracellular ROS level, which are the products of 6-OHDA catabolism (Meyerson et al. 1976; Minami et al. 1993). Salsolinol, applied as a racemic mixture, inhibited MAO activity in the rat brainstem and liver homogenates, and the inhibition was competitive to serotonin, a substrate of MAO type A, and non-competitive to benzylamine, a substrate of MAO type B (Meyerson et al. 1976). (*R*)-salsolinol inhibited MAO-A more potently than the (*S*)-enantiomer in vitro (Minami et al. 1993). The results were confirmed in vivo (Maruyama et al. 1993). The presence of hydroxyl groups at the sixth and seventh positions and substitution of a hydrogen group at the first position with a methyl or dihydroxybenzyl group are to be required for the inhibition, whereas the absence of a methyl group or presence of a carboxyl group at the first position, in addition to a methyl group, depletes the inhibitory activity (Bembenek et al. 1990; Thull et al. 1995).

(*RS*)-salsolinol also competitively inhibited COMT (Fig. 7) (Giovine et al. 1976). (*RS*)-salsolinol was O-methylated primarily at the 7-position, to form salsoline (l-methyl-7-methoxy-6-hydroxy-l,2,3,4-tetrahydroisoquinoline) (Collins and Origitano 1983). Salsoline can be accumulated in catecholamine nerve terminals of the brain and could act further as a false neurotransmitter (Cohen and Mytilineou 1982). The methylation of (*S*)-salsolinol should yield almost equivalent amounts of the two possible 6- and 7-methyl ethers, whereas the methylation of (*R*)-salsolinol should yield mostly (88%) 7-O-methyl derivative (Hötzl and Thomas 1997). Still, according to extensive literature, 1-methyl-1,2,3,4-tetrahydroisoquinoline (a non-catechol derivative) is regarded as one of the most potent neuroprotectants among tetrahydroisoquinolines in the brain (Fig. 7). Its neuroprotective effect did not induce the development of tolerance after chronic administration and might restore the function of dopamine neurons (Antkiewicz-Michaluk et al. 2001; Wąsik et al. 2016; Vetulani and Antkiewicz-Michaluk 2012; Peana et al. 2019). 1-Methyl-1,2,3,4-tetrahydroisoquinoline did not change the rate of total dopamine catabolism; it inhibited the monoamine oxidase (MAO)-dependent catabolic pathway and activated the catechol-O-methyltransferase (COMT)-dependent O-methylation. 1-Benzyl-1,2,3,4-tetrahydroisoquinoline, a compound considered as a highly neurotoxic (Fig. 7) (Antkiewicz-Michaluk et al. 2001, 2013; Yamakawa et al. 1999), produced the increase of the rate of dopamine metabolism with strong activation of the oxidative MAO-dependent catabolic pathway. It was also suggested that larger substituents at C1 may increase the neurotoxicity of tetrahydroisoquinolines (Wąsik et al. 2009).

Rodent models have been the most useful to study the occurrence, metabolism, and physiological function of (*RS*)-salsolinol (and other tetrahydroisoquinolines). Unfortunately, it is very difficult to pinpoint its exact physiological concentration since a whole array of chemically similar derivatives is associated with dopamine metabolism in the central (and peripheral) nervous system. Nervous system in general exhibits a great degree of cellular, structural, and chemical heterogeneity and neurotoxins can potentially affect any of its functional or structural components. So far, the neurotoxic properties of (*RS*)-salsolinol have been mostly laid stress; however, there is no agreement regarding its direct mechanism of action in the central and/or peripheral nervous system and no agreement regarding its ability to cross the blood-brain barrier either (Origitano et al. 1981). And surely, a great variety in test methods is needed to ensure its complete neurotoxicologic assessment. We decided to measure serum levels of TNFα and CRP as blood-based markers have the greatest potential for the translation of preclinical risk assessment to the human patient population (Tarrant 2010). TNFα is a major pro-inflammatory cytokine that exerts both homeostatic and pathophysiological roles in the central and peripheral nervous system. TNFα exerts its action via two receptors, TNF-R1 and TNF-R2, which are expressed in both glia and neurons. TNF-R1 is responsible for controlling the neuronal death. TNF-R2 contributes to neuroprotection due to his relation to T cell development and the proliferation (Takeuchi et al. 2006; Fregnan et al. 2012). High expression of TNFα at the site where neurological damage occurs which suggests that this pro-inflammatory cytokine is a mediator of neuronal injury (Leal et al. 2013). What is more, glial cells are supposed to release neurotoxic products not only into the brain parenchyma but also into the rest of the body by activating the endothelium of blood vessels and recruiting blood cells to the cerebral parenchyma. It was reported, for example, that plasma levels of TNFα remained increased even 1 year after intravenous MPTP (N-methyl-4-phenyl-1,2,3,6-tetrahydropyridine, a dopaminergic neurotoxin) administration in parkinsonian monkeys (Barcia et al. 2005). While CRP is an acute phase protein released by hepatocytes in response to increases in circulating inflammatory cytokines. Activation of complement is thought to contribute to the perpetuation of the inflammatory response and is implicated in neurodegenerative processes (Bonifati and Kishore 2007). CRP is also thought to interact with the blood-brain barrier—with increased paracellular permeability at a high dose that enables its entry into the central nervous system (CNS) and serves to induce reactive gliosis and impair CNS function (Hsuchou et al. 2012). According to some authors, CRP is a peripheral biomarker that reflects peripheral and central inflammation (and neuroinflammation) (Felger et al. 2018). Elevated systemic CRP levels have been noted in numerous conditions associated with peripheral and central neural damage as well as impaired neuroprotection, i.e., Alzheimer’s disease (Song et al. 2015; Kempuraj et al. 2017; Gabin et al. 2018), Parkinson’s disease (Sawada et al. 2015; Umemura et al. 2015; Qiu et al. 2019), depression (Felger et al. 2018), opioid use disorder (Wang et al. 2018), or following chimeric antigen receptor T cell (CAR-T) therapy in cancer (Santomasso et al. 2018; Wang and Han 2018). To justify our choice of markers, it should also be mentioned that both TNFα and CRP in serum have been evaluated in parkinsonian patients and correlated with motor deficits (Lindqvist et al. 2012; Eidson et al. 2017). In our experiment, TNFα serum levels but not CRP were only significantly different between animals implanted with ALZET osmotic mini-pumps. TNFα and CRP serum levels of salsolinol-treated rats were not significantly different from control animals, which should at least theoretically deny the neuroinflammatory and neurotoxic properties of (*RS*)-salsolinol, especially in the peripheral nervous system. Yet, methodological issues, such as route of administration, dose, and duration, should be taken into careful consideration. It needs to be noted that all applied doses were relatively small in comparison with other published in vivo experiments (for a summary, see Kurnik-Łucka et al. 2018). It remains unknown whether our results were mediated peripherally or centrally. We previously reported that (*RS*)-salsolinol was not detected (with the limit of detection set at 0.86 ng/l) in serum samples in a similar experimental model, which might suggest that (*RS*)-salsolinol did not reach the systemic blood (Kurnik et al. 2012). However, the blood samples were collected 24 h after the last delivery, and other related metabolites were not measured. Lee et al. (2010) demonstrated that a single administration of (*RS*)-salsolinol (10 μg) by gavage resulted in a significant elevation of rats’ plasma (*RS*)-salsolinol levels, which sharply declined to near basal levels by 14 h. The mean plasma concentrations of (*S*)- and (*R*)-salsolinol at 1 h after administration were 650 ± 46 and 614 ± 42 pg/ml, respectively. A single intake of 3 g banana (corresponding up to 75 μg of (*RS*)-salsolinol) also increased the plasma (*RS*)-salsolinol concentration. (*RS*)-salsolinol detected in the brain is likely to be derived from in situ synthesis [63], but some authors argue such a hypothesis (Sjöquist and Magnuson 1980; Song et al. 2006). Several authors have also reported that systemically administered (*RS*)-salsolinol is capable of altering behavior (Antkiewicz-Michaluk et al. 2000; Vetulani et al. 2001), which indirectly suggests that it could cross the blood-brain barrier. Yet, in our experiment, we did not observe any behavioral changes among animals. However, we previously observed an increase in the percentage of mean residual solid food in the stomach, suggesting reduced gastric emptying, which remains in agreement with the findings of Banach et al. (2006). We also reported a decrease in large intestine transit and water content of fecal matter as well as damage to myenteric neurons (Kurnik et al. 2015). Dopamine is indeed an important mediator of gastrointestinal secretion, absorption, and motility and is the predominant catecholamine neurotransmitter of both central and peripheral nervous systems. Intrinsic dopaminergic innervation was successfully identified in the bowel of mice and humans, and is believed to regulate motility and other bowel functions (Anlauf et al. 2003; Li et al. 2004, 2006). Therefore, there is a chance that (*RS*)-salsolinol might affect enteric dopaminergic neurotransmission. In vivo, the synthesis of (*RS*)-salsolinol was only demonstrated in the central nervous system (Naoi et al. 2002); however, recently, Villageliú et al. (2018) demonstrated that in vitro, *E. coli* can produce (*RS*)-salsolinol in the presence of dopamine with production enhanced in the presence of alcohol. Although this significant discovery needs an in vivo follow-up to explore whether (*RS*)-salsolinol production is a mechanism by which the microbiota may influence the host, it has already shed a new light on the (*RS*)-salsolinol’s metabolic pathways. Clearly, it is still too early to give a definite answer with regard to the neurotoxic profile of (*RS*)-salsolinol. Unaltered serum levels of TNFα and CRP together with our behavioral observations suggest that (*RS*)-salsolinol should not be neurotoxic in our in vivo model. But still, its potential neuroprotective properties need further in vivo examination especially due to the fact that it can be both delivered exogenously and synthesized endogenously.

Divergent results of several neuroprotection trials point out the limitations of transferring results from animal models and cell cultures to human pathology. It was suggested that only high concentration of tetrahydroisoquinolines (especially (*RS*)-salsolinol) and prolonged, probably continuous, exposure (endogenous and/or exogenous?) could lead to apoptotic cell death (DeCuypere et al. 2008; Możdżeń et al. 2015). Yet, Bettiol et al. (2015) reported that studies with a prospective design did not support any association between total alcohol intake and PD risk (with two studies finding an increased risk with a moderate alcohol consumption), and the case-control studies were more likely to find protective effects of alcohol on PD risk. Since (*RS*)-salsolinol is one of the many tetrahydroisoquinoline derivatives present in human brain, thus, it seems reasonable to hypothesize that not the absolute values but rather specific misbalance among them together with dopamine precursors and metabolites, possibly due to genetic and/or environmental instability, might be crucial in exerting its either neurotoxic and/or neuroprotective role.
